# Early postoperative changes in lung function after resection for lung cancer – do the complications have influence?

**DOI:** 10.1186/1749-8090-8-S1-O222

**Published:** 2013-09-11

**Authors:** M Ercegovac, D Subotic, V Zugic, R Jakovic, D Moskovljevic, S Bascarevic, N Mujovic, M Savic

**Affiliations:** 1 Clinic for Thoracic Surgery, Clinical Center of Serbia, Belgrade, Serbia; 2 Clinic for Pulmonology, Clinical Center of Serbia, Belgrade, Serbia; 3 Center for Physical Medicine and Rehabilitation, Clinical Center of Serbia, Belgrade, Serbia

## Background

The objective of this study was to investigate immediate postoperative lung function changes after resection for primary lung cancer in patients without and with postoperative complications.

## Method

Sixty patients undergoing surgical resection (sublobar, lobectomy, and pneumonectomy) for non-small cell lung cancer were included in a prospective, single institution study. Sex, age, stage and type of lung cancer, comorbidities, preoperative values of lung function tests including DLCO and exercise test were analyzed. Postoperative complications were classified as surgical, respiratory and cardiovascular. FEV1 and FVC were measured on postoperative days 1, 3 and 7, regardless of postoperative complications.

## Results

Study encompassed 60 patients (70% male, mean age 60.9±8.4 and 30% female, mean age 56.9±6.5). Mild degree of COPD was noted in 20% and moderate in 35% of patients, according to GOLD classification. All postoperative adverse events, not only major complications were recorded. Respiratory complications occurred in 20%, surgical in 41.7% and cardiac in 19% of patients. Measured postoperative values of FVC% and FEV1% on days 1, 3 and 7 after surgery shoved continuous improvement, with significant difference between the days of measurement, specially days 3 and 7. Values recorded on day 7 did not differ from ppo FEV1%. No difference in early postoperative trend of lung function recovery was noted between patients without and with postoperative complications. However, patients that developed respiratory complications showed significantly lower values of FEV1 and FVC on postoperative day 1.

## Conclusions

Early lung function after resection for lung cancer shows significant improvement despite of postoperative complications.

